# Results of a Feasibility and Acceptability Trial of an Online Smoking Cessation Program Targeting Young Adult Nondaily Smokers

**DOI:** 10.1155/2012/248541

**Published:** 2012-04-09

**Authors:** Carla J. Berg, Gillian L. Schauer

**Affiliations:** Department of Behavioral Sciences and Health Education, Emory University School of Public Health, 1518 Clifton Road NE, Atlanta, GA 30322, USA

## Abstract

Despite increases in nondaily smoking among young adults, no prior research has aimed to develop and test an intervention targeting this group. Thus, we aimed to develop and test the feasibility, acceptability, and potential effectiveness of an online intervention targeting college student nondaily smokers. We conducted a one-arm feasibility and acceptability trial of a four-week online intervention with weekly contacts among 31 college student nondaily smokers. We conducted assessments at baseline (B), end of treatment (EOT), and six-week followup (FU). We maintained a 100% retention rate over the 10-week period. Google Analytics data indicated positive utilization results, and 71.0% were satisfied with the program. There were increases (*P* < .001) in the number of people refraining from smoking for the past 30 days and reducing their smoking from B to EOT and to FU, with additional individuals reporting being quit despite recent smoking. Participants also increased in their perceptions of how bothersome secondhand smoke is to others (*P* < .05); however, no other attitudinal variables were altered. Thus, this intervention demonstrated feasibility, acceptability, and potential effectiveness among college-aged nondaily smokers. Additional research is needed to understand how nondaily smokers define cessation, improve measures for cessation, and examine theoretical constructs related to smoking among this population.

## 1. Introduction

Tobacco use is the number one preventable cause of death in the United States. Despite preventive efforts, approximately 46 million people or 19.9% of the US population smokes cigarettes [1]. Among American smokers, up to 33% smoke nondaily [2] or smoke between 1 and 29 days out of every 30 [3]. Nondaily smoking represents a common smoking pattern among young adults, with 19.9% reporting smoking less than 30 days per month [4].

Nondaily smokers suffer from significant smoking-related morbidity and mortality compared to individuals who have never smoked [5, 6]. According to the 2004 US Surgeon General's Report on the health consequences of smoking, individuals that are exposed to low levels of tobacco are still at risk for cardiovascular disease, lung and gastrointestinal cancers, lower respiratory tract infections, cataracts, compromised reproductive health, and osteoporosis [7]. In addition, smoking 5 or more days per month is associated with shortness of breath and fatigue and smoking at least 21 days per month is associated with symptoms of cough and sore throat [8]. Due to the health consequences of nondaily smoking, it is important to promote cessation, especially among young adult smokers since individuals that quit before the age of 30 will reduce their chances of dying prematurely from smoking-related diseases by more than 90 percent [7].

While a great deal of research has focused on developing cessation interventions for daily smokers, nondaily smokers are typically excluded from intervention studies because their level of smoking often does not meet the inclusion criteria for trials [9]. Unfortunately, nondaily smokers are less likely than heavier smokers to seek or receive treatment [10–12]. Nondaily smokers are significantly different in terms of their reasons for smoking and motivation to quit and thus require specific intervention strategies and messages. Nondaily smokers have also been shown to be more likely to be ready to quit in the next month, are more confident that they can quit, and are less likely to consider themselves to be addicted when compared to daily smokers [13]. While some report motivation to quit, they have difficulty quitting [14, 15].

From our prior research [16], we have identified several themes related to motivation to quit smoking, including wanting to avoid the stigma of being a smoker, particularly given that the majority of nondaily smokers do not consider themselves to be smokers [17]. Moreover, nondaily smokers reported concern about the opinions of friends, family, and significant others regarding smoking and concern about the impact of secondhand smoke exposure to others around them [16]. A number of nondaily smokers also reported only “smoking when they are drinking” and difficulty refraining from smoking while drinking [16]. Moreover, our research suggests that nondaily smokers more frequently use alcohol than daily smokers [18, 19]. Finally, nondaily smokers report a desire to quit smoking in order to avoid becoming addicted to cigarettes [16]. However, prior research indicated, that over 4 years, 50% of nondaily or occasional smokers in college continued to smoke, with one-third of these smokers progressing onto regular smoking [20].

Given these findings, we developed a four-module online intervention targeting nondaily smokers in the young adult population. The theoretical underpinnings for the intervention were drawn from (1) the Theory of Reasoned Action [21], which posits that behavior is the direct result of intention, which is, in turn, a function of the individual's attitude toward the behavior and his or her subjective norms about the behavior; (2) the Transtheoretical Model and Stages of Change [22], which states that change is a process of progressing through “stages of change” that related to measures of readiness. Based on our formative research and these theoretical frameworks, we developed an intervention targeting nondaily smoking in the college student population and subsequently tested the intervention for feasibility and acceptability as well as potential effectiveness.

## 2. Materials and Methods

### 2.1. Procedure

In October 2010, students at six colleges in the Southeast were recruited to complete an online survey assessing general health behaviors [23]. A random sample of 5,000 students at each school (with the exclusion of two schools who had enrollment less than 5,000) were invited to complete the survey (total invited *N* = 24,055). Of students who received the invitation to participate, 4,840 (20.1%) returned a completed survey. Eligibility requirements for this study included being between the ages of 18 and 30 years and being a nondaily smoker (i.e., smoking between 1 and 29 days of the past 30 days). We recruited 65 participants who met the eligibility criteria at the time of survey assessment. We enrolled 31 participants who met eligibility requirements, with the majority of participants that were not enrolled being excluded because they either increased their cigarette consumption to daily smoking or did not smoke in the past 30 days. The Emory University Institutional Review Board approved this study, IRB no. 00030631.

The intervention had a duration of four weeks and involved four weekly web-based sessions. Participants were asked to complete an online baseline assessment prior to the beginning of the intervention. During the intervention, participants were contacted via e-mail each week to request that they log into the intervention site. Upon logging in, participants were asked to complete a 7-day timeline followback reporting the number of drinks consumed on each day and the number of cigarettes they had on each day of the past 7 days. Upon clicking the “submit” button, participants were routed to the main intervention landing page. The website provided a graphical depiction of their daily alcohol consumption and daily cigarette consumption over the course of the intervention to date. In addition to this, they were presented with four modules over the four-week period, each of which included a video of 60 to 90 seconds in duration and a targeted message of approximately two brief paragraphs. The modules piloted in this feasibility and acceptability trial included (1) considering oneself a smoker versus the social stigma of being perceived as a smoker; (2) secondhand smoke exposure as a burden to others around you; (3) concurrent alcohol consumption and cigarette smoking; (4) likelihood of continued smoking or progression to regular smoking by graduation. These modules were selected given our prior research indicating the relevance of these four topics to nondaily smokers.

### 2.2. Measures

Participants completed assessments at baseline (Week 0), end of treatment (EOT; Week 4), and six-week followup (FU; Week 10). Participants received a $20 gift card for completing each of the assessments. We outline the data collected at each time point.

#### 2.2.1. Demographic Characteristics

 We assessed included participants' age, gender, and ethnicity. Ethnicity was categorized as non-Hispanic White, Black, or Other due to the small numbers of participants who reported other race/ethnicities.

#### 2.2.2. Process Evaluation Assessments

 We assessed participant retention over the course of the intervention. To assess the intervention components, we asked the questions listed in Table 1 at end of treatment. Response options were “yes” or “no” for questions with dichotomous answers or on a scale of 1 to 5 for questions using Likert scales, with a 5 indicating more favorable attitudes.

Data from Google Analytics [24] were also used to examine participant interaction with the website. We assessed average time spent on the site, number of participant visits, bounce rate, and number of page visits. The bounce rate indicates percentage of single-page visits or visits in which an individual left the site from the landing page, with a bounce rate of less than 35% being deemed as reasonable [25]. A high pages per visit average—of at least 3 pages—means visitors are interacting with site content, whereas a low average means visitors are viewing one page and quickly moving on to other sites [26].

#### 2.2.3. Alcohol Consumption

To assess alcohol consumption, participants were asked, “In the past 30 days, on how many days did you drink alcohol?” and “In the past 30 days, on how many of those days did you drink 5 or more drinks on one occasion?” These questions have been used to assess alcohol consumption and binge drinking, respectively, in the American College Health Association (ACHA) surveys, National College Health Risk Behavior Survey (NCHRBS), and Youth Risk Behavior Survey (YRBS), and their reliability and validity have been documented by previous research [3].

#### 2.2.4. Smoking Behaviors

To assess smoking status, participants were asked, “In the past 30 days, on how many days did you smoke a cigarette (even a puff)?” and “On the days that you smoke cigarettes, how many cigarettes do you smoke on average?” These questions have been used to assess tobacco use in the American College Health Association (ACHA) surveys, National College Health Risk Behavior Survey (NCHRBS), and Youth Risk Behavior Survey (YRBS), and their reliability and validity have been documented by previous research [3]. At baseline, participants were also asked to report the age at which they smoked their first whole cigarette and the age at which they started smoking regularly.

#### 2.2.5. Social Smoking

To assess social smoking, participants were asked, “In the past 30 days, did you smoke: mainly when you were with other people; mainly when you were alone, as often by yourself as with others, or not at all” [27]. This variable was dichotomized as “social smoking” (i.e., smoking mainly when with others) versus other responses.

#### 2.2.6. Identification of a Smoker

Participants were asked, “Do you consider yourself a smoker?” [17].

#### 2.2.7. Quit Attempts

At baseline, participants were asked, “During the past 12 months, how many times have you stopped smoking for one day or longer because you were trying to quit smoking?” [28]. This variable was dichotomized as having made at least one quit attempt in the past year versus not having made an attempt to quit. At baseline, they were also asked, “What is the longest time you were able to go without cigarettes in the past year? <24 hours; 1 to 7 days; 1 to 4 week; 1 to 3 months; 3 to 6 months; or 6 months to 1 year.” At end of treatment, they were asked, “What is the longest time you were able to go without cigarettes in the past four weeks? <24 hours; 1 to 7 days; 1 to 2 weeks; or 2 to 4 weeks.” At 6-week followup, participants were asked, “What is the longest time you were able to go without cigarettes in the past 10 weeks? <24 hours; 1 to 7 days; 1 to 2 weeks; 2 to 4 weeks; 4 to 6 weeks; 6 to 8 weeks; or 8 to 10 weeks.”

#### 2.2.8. Readiness to Quit Smoking

Readiness to quit was assessed by asking, “What best describes your intentions regarding quitting smoking: never expect to quit; may quit in the future, but not in the next 6 months; will quit in the next 6 months; will quit in the next month; and already quit” [29]. For the present study, this variable was categorized as already quit, intending to quit in the next 30 days, and all other responses.

#### 2.2.9. Concurrent Alcohol Use and Smoking

Participants were asked, “On a scale of 0 to 10, with 0 being ‘not at all difficult' and 10 being ‘extremely difficult,' how difficult is it for you to consume alcohol without smoking a cigarette?”.

#### 2.2.10. Perceived Harm of Smoking

 Participants were asked, “On a scale of 0 to 10 with 0 being ‘not at all harmful' and 10 being ‘extremely harmful,' how harmful to your health is smoking cigarettes?”.

#### 2.2.11. Beliefs about Secondhand Smoke (SHS)

 Participants were asked, “On a scale of 0 to 10 with 0 being ‘not at all harmful' and 10 being ‘extremely harmful,' how harmful to one's health do you think it is for people to be exposed to secondhand smoke?” and “On a scale of 0 to 10 with 0 being ‘no bother at all' and 10 being ‘extremely bothersome,' how much do you think secondhand smoke bothers those around you?”.

### 2.3. Data Analysis

Participant characteristics at baseline (Week 0), end of treatment (EOT; Week 4), and six-week followup (FU; Week 10) were summarized using descriptive statistics. Process evaluation assessments were summarized as well. Pairwise (within subjects) *t*-tests were conducted to examine differences in drinking, smoking-related variables, and psychosocial variables from baseline to EOT and from baseline to FU. Chi-squared tests examined categorical variables across time, comparing baseline to EOT and baseline to FU. SPSS 18.0 was used for all data analysis. Statistical significance was set at *α* = .05 for all tests. 

## 3. Results and Discussion

### 3.1. Results

Participants were 23.16 years of age on average (SD = 4.60), 80.6% (*n* = 25) female, 64.5% (*n* = 20) White, and 32.3% (*n* = 10) Black. Average age of having their first whole cigarettes was 17.35 years (SD = 3.62), and average age of starting smoking regularly was 19.03 years (SD = 4.08). At baseline, 15 (48.4%) reported having made a quit attempt in the past year, 22 (71.0%) were categorized as social smokers, and 15 (48.4%) considered themselves to be a smoker.

#### 3.1.1. Process Evaluation

 The intervention demonstrated 100% retention from baseline to EOT and to FU. Table 1 presents detailed data regarding the process evaluation of the study. Importantly, 54.9% of individuals gave scores of 4 or 5 regarding how helpful it was to track their smoking and alcohol over time, with 67.7% indicating that it was helpful to see a tailored graph of this information. Moreover, 71.0% reported being satisfied with the program (i.e., giving a score of 4 or 5), with 90.3% indicating that they would recommend the program to their friends who smoke.

 In addition, the utilization of the website per Google Analytics demonstrated positive results. Average time on the website per visit was 4 minutes and 2 seconds. We had a total of 379 visits over the course of the four weeks, averaging 3.05 visits per week per participant. There was a 28.5% bounce rate, and participants also were active on the website, with 4.80 pages per visit.

#### 3.1.2. Change in Smoking Behaviors and Attitudes

In terms of changes in average number of days of cigarette and alcohol consumption between baseline and the end of treatment (Week 4), no significant differences existed (see Table 2). However, significant decreases existed between baseline and followup (week 10) with regard to the number of days of alcohol consumption (*P* = .004), binge drinking (*P* = .02), and cigarette smoking (*P* < .001) as well as average CPD on smoking days (*P* = .003). In addition, there were increases in the number of people refraining from smoking for the past 30 days from baseline to EOT (*P* < .001) and to FU (*P* < .001), with 2 reporting no smoking in the past 30 days at EOT and 5 reporting no smoking in the past 30 days at FU. In terms of psychosocial factors, being quit for the past 30 days at EOT and FU was associated with confidence in quitting (EOT: 10.0 ± 0.00 versus 8.48 ± 1.50, *P* < .001; FU: 10.00 ± 0.00 versus 8.31 ± 1.49, *P* < .001). In addition, participants increased in their perceptions of how bothersome SHS is to others from baseline to EOT (*P* = .04) and to FU (*P* = .02); however, no other attitudinal variables were altered, and no attitudinal factors, either from baseline or as change scores, were related to smoking cessation outcomes at either EOT or FU.

At EOT, in addition to the two individuals that had not smoked in the past 30 days, 9 (29.0%) reported having reduced their smoking, with the average reduction of cigarette consumption on smoking days being 3.33 cigarettes (SD = 2.55) among those who reduced their smoking. At FU, in addition to the five individuals that had not smoked in the past 30 days, 17 (65.4%) reported having reduced their smoking, with the average reduction of cigarettes on smoking days being 1.73 cigarettes (SD = 1.35) among those who reduced their smoking.

Participants reported increases in readiness to quit from baseline to EOT (*P* = .001) and to FU (*P* = .003), with some individuals transitioning to quit status and some becoming ready to quit in the next 30 days, although this may be confounded by participants' definitions of the meaning “already quit smoking” (i.e., one of the response options for the readiness to quit smoking question) as compared to their reported number and frequency of cigarettes smoked. At the end of treatment (EOT), 6 (19.4%) reported having quit smoking per the assessment of readiness to quit, with two of these individuals not having smoked during the duration of the study and two individuals having smoked a total of 2 cigarettes over the course of the study (one had been abstinent over a week prior to the final assessment, and one had been abstinent over two weeks). Interestingly, one individual who reported being quit had smoked a total of 35 cigarettes over the course of the study on a total of 11 days, but had been abstinent for 3 days prior to the EOT assessment. This participant smoked 10 days of the past 30 days at baseline. Also, one individual who reported being quit at EOT reported smoking a total of 37 cigarettes over the course of the study on a total of 28 days without any abstinent days prior to the EOT assessment. Even more interestingly, 6 people had been abstinent for the entire fourth week, 5 had been abstinent during both the third and fourth weeks, 4 had been abstinent from the second to the fourth week, and 3 had reported no smoking in the first to fourth week. However, only four of these individuals selected the “already quit smoking” option.

### 3.2. Discussion

This is the first study to focus exclusively on developing and pilot testing an intervention targeting nondaily smoking among young adults. The current study is important for several reasons. First, it documents the feasibility and acceptability of an online smoking cessation intervention targeting nondaily smoking in the young adult population, as well as the acceptability and relevance of nondaily smoking cessation messages targeting this population. Second, it suggests the potential effectiveness of this intervention in effecting smoking reduction and cessation among young adult nondaily smokers. Finally, it highlights methodological issues related to evaluating a cessation intervention targeting nondaily smokers.

 In terms of feasibility, we were able to recruit the individuals who met eligibility for the current study; moreover, we were able to achieve 100% retention of our 31 participants over the four-week intervention period as well as over the six-week followup period. Our process evaluation assessments indicated that, on average, participants deemed the tracking and graphing of alcohol and cigarette consumption to be helpful and the messages and videos to be relevant and engaging. The majority also reported the messages and videos to have increased their motivation to and confidence in quitting smoking. Participants reported a high degree of satisfaction, and 90.3% reported that they would recommend the program to friends or family who smoke. Moreover, utilization of the website, per Google Analytics data, was appropriate, with a substantial amount of time spent on the website on average, repeated visits per participant per week over the course of the intervention, a reasonable bounce rate, and an appropriate pages per visit record [25, 26]. Thus, these data suggests the feasibility and acceptability of the intervention.

 Regarding changes in smoking behavior and attitudes over the course of the intervention and assessment period, our intervention demonstrated potential effectiveness, with a significant number of people reporting no smoking in the past 30 days at followup, six weeks after the intervention. However, we are unable to ascertain the proportion of nondaily smokers that would have been abstinent for the past 30 days without the intervention since, by definition, nondaily smokers do not smoke every day, or even every week. No research to date has assessed the rapid changes in nondaily smoking or the patterns of cigarette consumption that exist.

From baseline to six-week followup, we also documented a significant decrease in number of days of cigarette consumption, alcohol consumption, and binge drinking. A significant proportion of individuals also decreased the average cigarettes smoked per day during smoking days from the baseline to the six-week followup period. Prior research has documented that monitoring behavior in and of itself can lead to improvements in health behaviors [30]. However, the current study did not document significant changes over the four-week monitoring period. It is possible that participants continued to monitor their behavior either intentionally or unintentionally after the completion of the study. Alternatively, perhaps the natural course of the academic year had an impact on cigarette and alcohol consumption. A randomized controlled trial of this intervention would be able to address this issue.

 Finally, our intervention yielded promising results in terms of increasing participants' awareness regarding how bothersome nonsmokers around them may perceive SHS exposure to be. However, theoretical measures like motivation and confidence to quit smoking or attitudes about smoking were not significantly altered, nor were participants' appraisals of difficulty of drinking alcohol without smoking. Furthermore, participants did not demonstrate significant changes in perceived harm of smoking or SHS exposure. However, participants perceive a high level of harm of smoking and SHS at baseline; thus, this might reflect a ceiling effect for perceived harm. Moreover, it may also be that individuals who perceived a high level of harm were more likely to enroll in this study, which should be examined in subsequent research. Two factors may have influenced the effect of the intervention on perceived bother of SHS to others. First, baseline ratings on this assessment of perceived bother of SHS were lower than perceived harm and thus were less likely to have a ceiling effect. Second, one of the modules explicitly focused on the bother of SHS exposure to nonsmokers, whereas the other variables (addressing specific skills related to refraining from smoking while drinking or perceived harm of smoking or SHS exposure) were less central to other intervention messages. The fact that smoking behavior changed despite the fact that attitudinal, motivational, and perceived harm measures were not altered indicates that theoretical and psychosocial constructs that typically predict behavior may operate differently in nondaily smokers. Among regular or daily smokers, motivation and confidence to quit [31–33] as well as perceived harm of smoking [34, 35] predict smoking cessation. However, among our sample of nondaily smokers, confidence in quitting was the only baseline factor that was associated with EOT and FU cessation. It is possible that other factors are less central to behavior change in nondaily smokers given the less established pattern of the behavior and/or the cognitive dissonance that might exist around the behavior (e.g., nondaily smokers often do not consider themselves to be smokers [17]). Our findings suggest the need to examine the relative contribution of these varied factors to the process of smoking cessation and reduction among nondaily smokers.

 Despite relatively few changes in attitudinal variables in general, participants demonstrated trends in increased readiness to quit smoking. However, a critical finding from this research relates to the varied definitions and perceptions that nondaily smokers have of being “quit.” In light of the inconsistencies in reported “quits” as compared to reported smoking, further examination of nondaily smokers' conceptualization of smoking status and smoking cessation is warranted. Furthermore, our study highlights the need for additional qualitative research to better understand how nondaily smokers define their own cessation and quantitative research to further define measurements for smoking cessation among nondaily smokers. It is critical to strive for consistency of research in this area and for clear articulation when gathering cessation status from nondaily smokers.

### 3.3. Limitations

 A number of limitations should be considered when interpreting these results. First, this was a small sample, limiting the overall power of the study. However, despite the sample size, a number of significant and important findings were detected related to cessation in nondaily smokers, a population that has not been extensively studied. In addition, the sample was drawn exclusively from southeast colleges, which limits generalizability to other parts of the country or to other groups of young adults. On a related note, our sample was largely female despite the fact that young adult males have a higher rate of smoking [36]. Greater efforts are needed to recruit and enroll young adult male nondaily smokers into interventions targeting nondaily smoking. Furthermore, no control condition was included to determine whether or not changes were due to the intervention or simply to the changing smoking patterns of nondaily smokers. Future research should include a control arm and be tested for efficacy among a larger sample of nondaily smokers in the young adult population.

## 4. Conclusions

This study suggests that an online intervention targeting factors specific to nondaily smoking is acceptable to a college-aged nondaily smoker population and that individuals will participate and engage in such an intervention. Additional research is needed to better understand how nondaily smokers define cessation and to establish and improve measurement standards for cessation among this population.

## Figures and Tables

**Table 1:** Process evaluation outcomes at Week 4.

Variable	Mean (SD) or *N* (%)
Participant assessments	

How helpful was it to track your own smoking and alcohol use over time?	3.74 (0.77)
How helpful was it to see a graph of your smoking/drinking level during the program?	3.81 (1.01)
*Would you recommend keeping this in the program?	30 (96.8)
How much of the reading material did you read?	3.58 (1.20)
How relevant was the material to you?	3.42 (1.20)
How interesting or engaging were the messages?	3.55 (1.12)
*Did the messages increase your motivation to quit smoking?	19 (61.3)
*Did the messages increase your confidence in being able to quit smoking?	21 (67.7)
*Would you recommend keeping these messages in the quit smoking program?	30 (96.8)
How much of the videos did you watch?	3.52 (1.26)
How relevant was the video content to you?	3.65 (1.36)
How interesting or engaging were the videos?	3.62 (1.20)
*Did the videos increase your motivation to quit smoking?	20 (64.5)
*Did the videos increase your confidence in being able to quit smoking?	18 (58.1)
*Would you recommend keeping the videos in the quit smoking program?	31 (100.0)
Overall, how satisfied were you with the program?	4.16 (0.93)
How much influence did the program have on your motivation to quit?	3.39 (1.25)
How much influence did the program have on your confidence to quit smoking?	3.32 (1.24)
*Would you recommend participating in this program to your friends who are smoking?	28 (90.3)

Web utilization	

Average time on the site	4: 02
Total visits	379
Bounce rate	28.5%
Number of pages per visit	4.80

Note. Scale items are on a scale of 1 to 5 with higher ratings indicating more favorable attitudes.

* % reporting “yes.”

**Table 2:** Bivariate analyses comparing Week 0 to Week 4 and Week 10 factors.

Variable	Week 0 (baseline)	Week 4 (End of Tx)	*P* value	Week 10 (6-week FU)	*P* value
	Mean (SD) or *N* (%)	Mean (SD) or *N* (%)		Mean (SD) or *N* (%)	
Number of days of drinking, past 30 days (SD)	9.97 (7.38)	11.00 (8.01)	.38	7.55 (6.92)	.004
Number of days of binge drinking, past 30 days (SD)	3.54 (4.79)	3.29 (3.60)	.74	2.32 (2.81)	.02
Number of days of smoking, past 30 days (SD)	14.83 (10.43)	14.45 (10.21)	.54	10.87 (10.92)	<.001
Smoked in the past 30 days (%)	31 (100.0)	29 (93.5)	<.001	26 (83.9)	<.001
Ave. CPD on smoking days (SD)	3.00 (2.24)	2.80 (1.87)	.37	2.29 (1.87)	.003
Longest abstinence in the past year (%)					
<24 hours	—	—	—		—
1 to 7 days	8 (25.8)				
1 to 4 week	3 (9.7)				
1 to 3 months	8 (25.8)				
3 to 6 months	9 (29.0)				
6 months to 1 year	3 (9.7)				
Longest abstinence in the past four weeks (%)					
<24 hours	—	—	—		—
1 to 7 days		22 (71.0)			
1 to 2 weeks		2 (6.5)			
2 to 4 weeks		7 (22.6)			
Longest abstinence in the past 10 weeks (%)					
<24 hours	—	—	—	1 (3.2)	—
1 to 7 days				10 (32.3)	
1 to 2 weeks				4 (12.9)	
2 to 4 weeks				8 (25.8)	
4 to 6 weeks				3 (9.7)	
6 to 8 weeks				0 (0.0)	
8 to 10 weeks				5 (16.1)	
Readiness to quit in next 30 days (%)			.001		.003
No	26 (83.9)	20 (64.5)		21 (67.7)	
Yes	5 (16.1)	5 (16.1)		6 (19.4)	
Already quit	—	6 (19.4)		4 (12.9)	
Confidence in quitting (SD)	8.58 (1.50)	8.16 (2.30)	.28	8.48 (1.69)	.75
Motivation to quit (SD)	6.52 (3.38)	6.23 (3.12)	.42	7.06 (3.24)	.39
Difficulty drinking without smoking (SD)	4.74 (3.83)	4.80 (3.63)	.91	4.81 (3.62)	.92
Perceived harm of smoking (SD)	8.77 (2.09)	8.75 (1.98)	.93	9.00 (1.43)	.57
Perceived harm of secondhand smoke (SD)	8.67 (1.83)	8.52 (2.11)	.55	8.48 (2.04)	.47
Perceived bother of secondhand smoke (SD)	6.35 (3.31)	7.48 (2.11)	.04	7.52 (2.59)	.02
